# Refractory Relapsing–Remitting Adult‐Onset Still's Disease in an Adolescent Female: A Rare Case Report

**DOI:** 10.1002/ccr3.73290

**Published:** 2026-08-04

**Authors:** Tasnim Nafian, Mohd. Mujtaba Akib Bhuiyan, Kazi Ali Aftab, Khaled Mahbub Murshed, Md. Abul Kalam Azad

**Affiliations:** ^1^ Department of Internal Medicine Bangladesh Medical University Dhaka Bangladesh

**Keywords:** adult‐onset Still's disease, biologic therapy, case report, interleukin‐6, relapsing–remitting, tocilizumab, Yamaguchi criteria

## Abstract

Adult‐onset Still's disease (AOSD) is a rare systemic autoinflammatory condition that presents with symptoms such as episodic fevers, a transient rash, inflammatory arthritis, and significant systemic inflammation. Diseases that have a relapsing–remitting clinical course can be challenging to diagnose and treat. We present a case of an 18 year old female with multiple flares over several years requiring stepwise escalation from conventional disease‐modifying antirheumatic drugs (DMARDs) to targeted biologic therapy, highlighting the challenges of achieving durable remission. Our patient presented with recurrent episodes of high‐grade fever (up to 40°C), evanescent salmon‐pink rash, bilateral symmetrical inflammatory polyarthritis involving the wrists, metacarpophalangeal (MCP), proximal interphalangeal (PIP) joints, and knees with mild splenomegaly. After ruling out infectious, malignant, and other autoimmune diseases via biochemical, serological, autoimmune testing and biopsy, she was diagnosed with AOSD on the basis of the Yamaguchi criteria. Despite receiving treatment with corticosteroids and conventional disease‐modifying antirheumatic drugs (DMARDs), including methotrexate, she suffered from multiple relapses, indicating a polycyclic disease pattern. The introduction of biologic therapy with tocilizumab provided temporary remission. However, her flares returned during the tapering of steroids, necessitating an increase in the dosage and the use of pulse corticosteroid therapy during the current admission. This case report illustrates the relapsing–remitting nature of AOSD. Escalation to IL‐6 receptor inhibition (tocilizumab) is required in DMARDs refractory relapsing AOSD, with dose optimization to achieve sustained remission of disease activity of our patient.

## Key Clinical Message

The polycyclic disease pattern frequently coincides with corticosteroid dose reduction, reflecting suppression of the underlying inflammatory process rather than its complete elimination. Escalation to IL‐6 receptor inhibitor (tocilizumab) is required in DMARD‐refractory relapsing AOSD, with dose optimization to achieve sustained remission of disease activity of our patient.

**Abbreviations**
ANA: anti‐nuclear antibody
ANCA: anti‐neutrophil cytoplasmic antibody (p‐ANCA: perinuclear c‐ANCA: cytoplasmic)
AOSD: adult‐onset Still's disease
CRP: C‐reactive protein
CXR: chest X‐ray
DMARD: disease‐modifying antirheumatic drug
MCP: metacarpophalangeal
PIP: proximal interphalangeal
RA: Rheumatoid arthritis
RF: rheumatoid factor
SLE: Systemic lupus erythematosus

## 1 Introduction

AOSD is a rare systemic autoinflammatory condition. It is characterized by episodes of high grade fever, transient salmon‐pink rashes, inflammatory polyarthritis, and increased levels of inflammatory markers [1]. In 20%–25% cases, it can cause significant morbidity due to systemic inflammation and chronic joint damage [2]. AOSD is linked to an imbalance in innate immunity which leads to an excessive production of inflammatory cytokines, especially IL‐1, IL‐6, and IL‐18 [3]. Increasing evidence suggests that the classification of AOSD is fundamentally a cytokine driven condition that falls within the broader category of autoinflammatory disorders [4].

Clinically, AOSD may follow three patterns: monocyclic, polycyclic (relapsing–remitting), and chronic articular disease [5]. While corticosteroids remain first‐line therapy, many patients may require conventional DMARDs such as methotrexate for steroid‐sparing effects [2]. In refractory cases, biologics targeting IL‐1 and IL‐6 pathways have demonstrated significant efficacy [6]. Tocilizumab, an IL‐6 receptor inhibitor, has shown benefit in randomized controlled trials and observational studies in patients with systemic and articular manifestations of refractory AOSD [7, 8]. We present a case of relapsing–remitting AOSD requiring escalation to IL‐6 inhibitor therapy.

## 2 Case Presentation

### 2.1 Case History

An 18 year old Asian Muslim female patient with no known history of hypertension or diabetes presented with complaints of multiple joint pain and fever for 1 month, along with rash of the same duration.

Her illness had initially begun approximately 30 months before the current presentation with a sudden onset generalized erythematous pruritic rash sparing her face. The rash improved partially with topical and oral dermatological treatment. A few days later, she developed severe generalized body ache, more pronounced in the morning, significantly limiting her mobility. She was admitted to a tertiary hospital with high grade fever (up to 104°F), without any localizing symptom.

She initially received IV ceftriaxone for 7 days. Due to a persistent fever, IV meropenem was given but was discontinued after suspecting a drug reaction. Dengue NS1 antigen was positive and she was managed supportively. Persistent fever led to re‐admission to a private hospital. IV amikacin was initiated but was later discontinued due to the occurrence of a drug‐induced rash. Empirical oral prednisolone was subsequently prescribed, resulting in transient symptomatic improvement.

Several months later, she consulted an internist. Other than her symptoms, investigations revealed leukocytosis with 85% neutrophil, high ferritin level, negative ANA & RF, and mild splenomegaly on USG. Based on the Yamaguchi criteria, she was diagnosed with AOSD. A naproxen challenge test resulted in dramatic clinical improvement. She was prescribed hydroxychloroquine and oral prednisolone, with initial symptomatic relief.

Approximately 6 months later, she traveled to India for further evaluation. The diagnosis remained the same and hydroxychloroquine was replaced by oral methotrexate (15 mg weekly). However, recurrent fever, rash, and polyarthralgia persisted. In the following month, the methotrexate dose was increased, along with the continuation of corticosteroids and analgesics, and she remained symptom free for nearly 8 months. Tofacitinib was introduced following her relapse but discontinued after 7 days due to pruritus. Hydroxychloroquine was then reintroduced without significant improvement. Over the ensuing months, the patient continued to experience recurrent episodes of fever, rash, and inflammatory joint pain, requiring steroid dose adjustment.

After 2 months, the patient was re‐admitted with symptoms including fever, rash, polyarthritis, lymphadenopathy, and mild splenomegaly. The hematological report indicated neutrophilic leukocytosis with a CRP level of 117.7 mg/dL (reference range < 5 mg/L), serum ferritin level of 28,533 ng/mL (reference range 22–120 ng/mL), and procalcitonin measuring 0.33 ng/mL (reference range < 0.05 ng/mL). Elevated liver enzymes were also noted, while ANA was negative. Blood and urine cultures showed no growth. Following a thorough evaluation, she was diagnosed with a flare of Adult Onset Still's Disease (AOSD). The treatment regimen included subcutaneous methotrexate at 15 mg weekly (equivalent to 60 IU on an insulin syringe scale of a 25 mg/mL formulation) weekly and oral prednisolone at 30 mg per day, which was tapered gradually.

After approximately 2.5 months of remission, she experienced fever, rash, and joint pain of the same pattern during the tapering of corticosteroids, indicating a relapsing–remitting disease pattern. Consequently, treatment was modified to IV tocilizumab at 4 mg/kg monthly, SC methotrexate at 20 mg weekly, and oral prednisolone at 15 mg with subsequent tapering. She remained symptom‐free for about three months. However, symptoms recurred as the steroids were tapered down.

During the current admission, she presented with a high‐grade intermittent fever for 1 month, with temperatures reaching up to 104°F, bilateral symmetrical inflammatory polyarthritis affecting the wrists, MCP, PIP, and knee joints accompanied by morning stiffness after remaining symptom‐free for 3 months. An evanescent salmon‐pink rash developed during the febrile episodes.

She gave no prior history of chronic illness or autoimmune disease before mid‐2023. No significant family history or psychosocial history of relevance. No genetic workup was undertaken because the clinical presentation and laboratory findings were considered sufficient for disease establishment and the results were not expected to alter her management.

### 2.2 Physical Examination

The patient appeared ill with signs of anemia. A macular rash was present over the limbs, chest, and abdomen (Figure 1). Affected joints were tender, raised local temperature; range of motion was preserved and no deformity was observed. Other systemic examinations were unremarkable.

Chronological clinical timeline of disease course and therapeutic interventions in Table 1.

**FIGURE 1:**
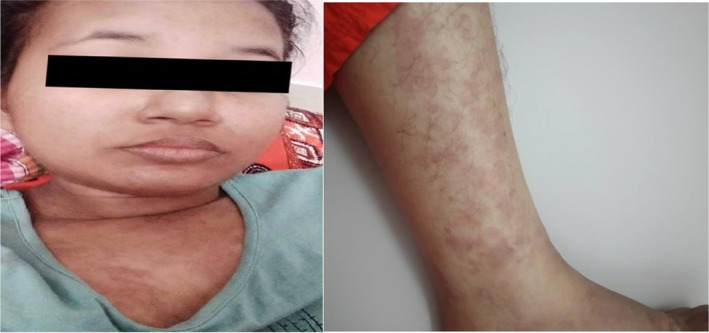
Evanescent salmon‐pink rash present during fever, which disappeared after subsidence of fever.

**TABLE 1:** Chronological clinical timeline of disease course and therapeutic interventions.

Date	Clinical events & management
~30 months prior to most recent admission	Sudden‐onset erythematous pruritic rash, severe generalized body ache, high‐grade fever (40°C); admitted to tertiary hospital; received IV ceftriaxone then IV meropenem (discontinued); empirical oral prednisolone started.
~26 months prior to most recent admission	Evaluated by internist; investigations revealed leukocytosis (85% neutrophils), ferritin 14,740 ng/mL (reference range 22–120 ng/mL), negative ANA & RF, mild splenomegaly on USG. AOSD diagnosed per Yamaguchi criteria. Naproxen challenge → dramatic improvement. Prescribed hydroxychloroquine and oral prednisolone.
~20 months prior to most recent admission	Traveled to India for further evaluation; diagnosis confirmed. Hydroxychloroquine replaced by oral methotrexate (MTX) 15 mg/week. Persistent fever, rash and polyarthralgia continued.
~18 months prior to most recent admission	MTX dose escalated; corticosteroids and analgesics continued.
~10 months prior to most recent admission	Tofacitinib was introduced and discontinued after 7 days due to pruritus. Hydroxychloroquine reintroduced but no significant improvement.
~8 months prior to most recent admission	Re‐admitted with AOSD flare. CRP 117.7 mg/dl, ferritin 28,533 ng/ml. SC methotrexate 15 mg/week and prednisolone 30 mg/day initiated (tapered gradually).
~4 months prior to most recent admission	Relapse during steroid tapering (approximately 2.5 months remission). Therapy escalated to IV tocilizumab 4 mg/kg/month + SC MTX 20 mg/week + prednisolone 15 mg. Symptom‐free for approximately 3 months.
Most recent admission (Day 1)	Re‐admitted with 1‐month history of high‐grade intermittent fever (40°C), bilateral symmetrical polyarthritis (wrists, MCP, PIP, knees), morning stiffness, and evanescent salmon‐pink rash with febrile episodes. Her CRP > 200 mg/dL (reference range < 5 mg/dL), ferritin 43,564 ng/mL (reference range 22–120 ng/mL), WBC 34 × 10^9^/L (refence range 7‐11 × 10^9^/L) (97% neutrophils). Escalated to tocilizumab 8 mg/kg/month + SC MTX 25 mg/week + pulse methylprednisolone for 3 days, then oral prednisolone 30 mg with tapering.

## 3 Methods (Differential Diagnosis, Investigations, Treatment)

Her diagnosis is refractory relapsing–remitting AOSD. The differentials included infections (sepsis, infective endocarditis), malignancies (lymphoma, leukemia), and other connective tissue diseases (SLE, RA, systemic vasculitis), which were ruled out based on clinical assessment, laboratory investigations, microbiological studies, imaging findings, and bone marrow findings.

### 3.1 Laboratory Investigations and Imaging

Investigations performed during the diagnosis and during the most recent admission are summarized in Table 2.

**TABLE 2:** Investigation summary of the patient.

Investigation	Diagnostic evaluation	Most recent admission	Reference range
Hematological findings			
Hb (gm/dl)	7.8	9.5	12.00–15.00
ESR (mm/1^st^ hr)	45	100	0–10
WBC (×10^9^/L)	16.5	34	4.0–11.0
Neutrophil (%)	85	97	40–80
Platelet (×10^9^/L)	340	350	150–450
PBF	Microcytic hypochromic anemia with neutrophilic leukocytosis	Dimorphic anemia with neutrophilic leukocytosis	
CRP (mg/dl)	172	> 200	< 5.00
Culture and sensitivity findings			
Blood C/S	Negative	Negative	
Urine C/S	Negative	Negative	
Biochemical findings			
S. Ferritin (ng/ml)	14,740	43,564	22–120
Procalcitonin (ng/ml)	0.132	0.7	< 0.05
AST (U/L)	42	54	10–35 (in female)
Fibrinogen (mg/dl)		375	200–400
LDH (IU/L)	780		135–225
CPK (U/L)		29	22–145
Immuno‐serological and serological findings			
HBsAg, Anti HCV, Anti HIV	Negative	Negative	
SLE panel (ANA, Anti dsDNA, Anti Sm)	Negative	Negative	
p‐ANCA	Negative		
c‐ANCA	Negative		
Serum C3 (g/L)		2.09	0.90–1.80
Serum C4 (g/L)		0.56	0.10–0.40
RF	Negative	Negative	
Bone marrow and imaging findings			
Bone Marrow	Hyperactive marrow with myeloid hyperplasia	Suggestive of myeloid & erythroid hyperplasia	
CXR	Normal	Normal	
USG of W/A	Mild splenomegaly		
Echo	Mild AR Pericardial effusion	Mild AR Pericardial effusion	

### 3.2 Trephine Biopsy and Histopathology

Microscopic appearance: Sections show cores of spongy bone. Bone marrow is hypercellular and expanded with lymphohistiocytic cells. The erythropoiesis is active and granulopoiesis is depressed. Megakaryocytes are seen (Figure 2).

**FIGURE 2:**
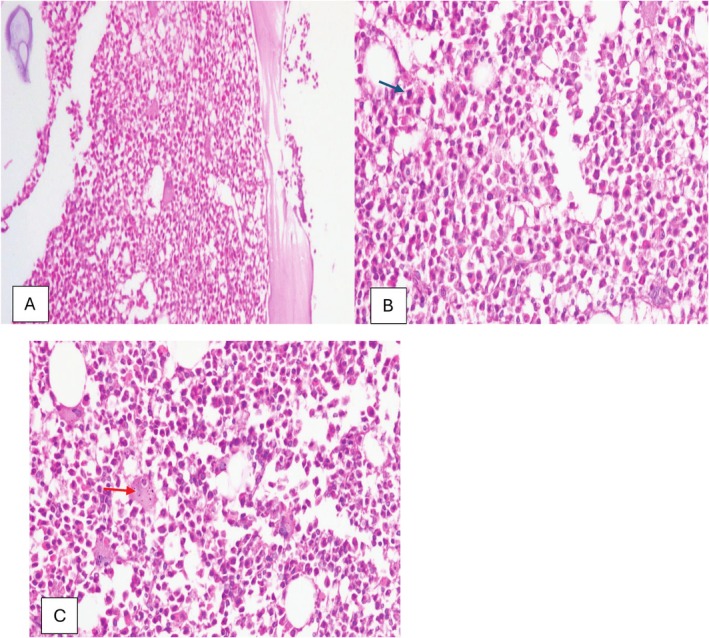
Histopathology of bone marrow trephine biopsy showing hypercellular marrow in low power objective (A); plasmacyte (blue arrow head) and histiocyte (red arrow head) in high power objective (B, C); with hemophagocytosis (red arrow head) (C).

Diagnosis of AOSD is itself challenging due to its nonspecific presentation. Since it is a disease of exclusion and this region has a high burden of infectious diseases, her diagnosis was delayed for 5 months. Additionally, due to limited access to specialized biomarkers in resource‐constrained settings, diagnosis was supported by fulfillment of the Yamaguchi criteria (Table 3), together with compatible clinical features and exclusion of differentials.

**TABLE 3:** Yamaguchi criteria fulfilled for diagnosis.

Major Criteria	
Fever ≥ 39 ▪ C for ≥ 1 week	Present
Arthralgia ≥ 2 weeks	Present
Typical rash	Present
Leukocytosis with neutrophilia (≥ 80%)	Present
Minor criteria	
Sore throat	Absent
Lymphadenopathy or splenomegaly	Mild splenomegaly
Liver dysfunction	High LDH
Negative RA and ANA	Both negative

The patient was initially treated with oral prednisolone and hydroxychloroquine, after which oral methotrexate 15 mg weekly was introduced and later increased due to ongoing symptoms. During disease relapse, treatment was intensified with SC methotrexate (15 mg weekly, then 20 mg weekly) and oral prednisolone 30 mg daily with tapering. Due to recurrent flares, IV tocilizumab 4 mg/kg monthly was initiated. During the most recent severe flare, treatment was further escalated to IV tocilizumab 8 mg/kg monthly, SC methotrexate 25 mg (100 IU based on insulin syringe pump of a 25 mg/mL formulation) weekly pulse IV methylprednisolone (500 mg daily for three days) with oral corticosteroids 30 mg with tapering.

## 4 Results and Conclusion (Outcome and Follow‐Up)

Following the escalated treatment regimen, the patient achieved significant clinical improvement during the current admission. Fever resolved, rash subsided, and joint symptoms improved considerably. Biochemically, the WBC count normalized and CRP declined to < 10 mg/dL, indicating suppression of the systemic inflammatory response.

The patient was discharged on IV tocilizumab 8 mg/kg monthly, SC methotrexate 100 IU weekly, and a tapering dose of oral prednisolone. Close outpatient follow‐up was planned, with monitoring of inflammatory markers (CRP, ESR, serum ferritin), complete blood count to assess disease activity and treatment response. Our patient demonstrated good compliance and treatment was well tolerated during hospitalization and early follow up over phone. No significant adverse effects were noted during treatment course.

## 5 Discussion

This case report provides a detailed description of resistant relapsing–remitting course of AOSD and highlights the diagnostic challenges along with therapeutic complexity. AOSD is a rare systemic autoinflammatory disorder and a diagnosis of exclusion, established only after ruling out infectious, hematological, and other autoimmune conditions. The diagnosis was supported by fulfillment of the Yamaguchi classification criteria, which have a reported sensitivity of approximately 96.2% when five or more criteria are present. Although originally developed as classification rather than diagnostic criteria, they are widely used to support the diagnosis of AOSD after exclusion of alternative causes [9]. In our patient, diagnosis is supported by these criteria. Macrophage activation syndrome (MAS) is a critical complication of AOSD. Although the patient's ferritin level was markedly elevated, she did not fulfill the diagnostic criteria for MAS because cytopenias, hypofibrinogenemia, and significant hepatic dysfunction were absent [10]. The long‐term course of AOSD is diverse [11]. Observational studies have identified three principal clinical pattern [1, 5]. The polycyclic course is demonstrated in our patient. Her symptoms involve repeated inflammatory exacerbations with periods of remission [5]. Reports from different regions, including individual case analyses and cohort studies, describe similar recurrent presentations requiring repeated modification of therapy [12, 13]. Several longitudinal studies indicate that disease flare frequently coincides with a reduction of corticosteroid dosage [4, 5]. This pattern suggests that clinical improvement during steroid therapy may only represent suppression. The 2.5‐year history of steroid‐dependent recurrence in our patient follows this globally documented disease behavior. Excessive production of pro‐inflammatory cytokines—particularly IL‐1, IL‐6, and IL‐18—plays a central role in the pathogenesis of AOSD [3]. IL‐6 contributes to systemic manifestations. It induces fever, stimulates hepatic acute‐phase protein synthesis, promotes anemia of chronic disease, and sustains joint inflammation [2]. Glucocorticoids exert broad immunosuppressive effects; however, they do not selectively neutralize upstream cytokine signaling. That is why tapering of glucocorticoids causes reactivation of inflammatory pathways. This biological mechanism reasonably explains the cyclical relapse observed in our patient during steroid reduction Current treatment strategies for AOSD are guided by disease severity and predominant clinical manifestations. NSAIDs may be considered in patients with mild disease and limited systemic involvement, whereas systemic corticosteroids remain the cornerstone of treatment for moderate‐to‐severe disease and provide rapid symptom control. Conventional DMARDs, particularly methotrexate, are commonly employed as steroid‐sparing agents and are especially useful in patients with persistent articular manifestations [2, 7, 14, 15]. Adjunctive glucocorticoids remain important for achieving rapid control of active inflammation in moderate‐to‐severe disease, whereas pulse intravenous methylprednisolone is generally reserved for severe disease flares, rapidly progressive systemic manifestations, impending organ dysfunction, or suspected macrophage activation syndrome [15]. In our case, despite methotrexate and prolonged corticosteroid exposure, cytokine blockade is required to achieve sustained control. Advances in immunopathogenic understanding have led to the use of biologic agents targeting specific cytokines. IL‐1 inhibitors have demonstrated effectiveness, particularly in systemic manifestations [6]. Current expert recommendations generally favor IL‐1 blockade (e.g., anakinra or canakinumab) as the preferred first‐line biologic strategy in patients with predominant systemic AOSD because IL‐1 plays a central role in disease pathogenesis. However, IL‐6 receptor inhibitors such as tocilizumab remain effective alternatives when IL‐1 inhibitors are unavailable, contraindicated, poorly tolerated, or inaccessible because of cost and local availability [15]. A randomized controlled trial demonstrated that tocilizumab significantly improved clinical outcomes in glucocorticoid‐refractory AOSD [8]. Case reports published in international journals, including American Journal of Case Reports and Medicine (Baltimore), have similarly described successful biologic intervention in recurrent AOSD presentations [14, 15]. Clinical trials have commonly utilized intravenous doses of 8 mg/kg every 2–4 weeks [8]. In our patient, initial therapy with tocilizumab at 4 mg/kg achieved only temporary stabilization. Escalation to 8 mg/kg monthly dosing, together with pulse methylprednisolone, resulted in sustained clinical improvement. Tocilizumab is generally well tolerated; however, clinicians should remain vigilant for adverse effects including serious infections, neutropenia, thrombocytopenia, elevated liver enzymes, gastrointestinal perforation, and dyslipidemia [16]. Despite its efficacy, access to biologic therapy remains challenging in many resource‐limited settings because of high treatment costs, limited availability, and reimbursement constraints [15, 17]. These factors may delay initiation of optimal therapy and influence treatment selection. In resource‐constrained environments, treatment decisions often require balancing evidence‐based recommendations against local availability and affordability. This consideration was particularly relevant in our setting and may explain differences between real‐world practice and treatment regimens employed in clinical trials [18].

This case highlights the challenges of managing relapsing–remitting AOSD in resource‐limited settings and underscores the need for individualized treatment strategies based on disease severity, treatment response, and access to advanced therapies.

## 6 Conclusion

Relapsing–remitting AOSD remains a therapeutic challenge which requires proper investigation & multidisciplinary approach for management of the patient. In our patient, corticosteroid therapy only causes temporary disease suppression. Timely escalation to targeted biologic therapy is required with personalized dose management when conventional DMARDs proved to be insufficient. This helps to improve long‐term outcomes of our patient and reduce her dependence on corticosteroids.

## Author Contributions

**Kazi Ali Aftab:** conceptualization, supervision, validation, resources, writing – review and editing. **Khaled Mahbub Murshed:** formal analysis, investigation, validation, writing – review and editing. **Md. Abul Kalam Azad:** writing – review and editing, project administration, supervision, validation, resources. **Tasnim Nafian:** conceptualization, data curation, writing – original draft, investigation, visualization, formal analysis. **Mohd. Mujtaba Akib Bhuiyan:** investigation, data curation, validation, writing – review and editing.

## Funding

The authors have nothing to report.

## Disclosure

Patient Perspective: Our patient shared serious concern about recurrent fever, rash, and joint pain that repeatedly affected her daily lifestyle and academic progression. She cooperated with us throughout her diagnostic evaluation. She agreed to follow the current treatment according to her understanding of the disease nature hoping this will help to maintain disease control.

## Ethics Statement

This case report was exempted from formal ethical review as per in‐situational guidelines for case reports. Written informed consent was obtained from the patient to publish this case report and accompanying images.

## Consent

Written informed consent was given by the patient along with accompanying images. A copy of the written consent is available for review by the Editor‐in‐chief of this journal.

## Conflicts of Interest

The authors declare no conflicts of interest.

## Data Availability

All data generated or analyzed during this study are included in this published article.
